# A bibliometric analysis of artificial intelligence research in critical illness: a quantitative approach and visualization study

**DOI:** 10.3389/fmed.2025.1553970

**Published:** 2025-03-04

**Authors:** Zixin Luo, Jialian Lv, Kang Zou

**Affiliations:** ^1^The First Clinical Medical College, Gannan Medical University, Ganzhou City, Jiangxi, China; ^2^Department of Critical Care Medicine, The First Affiliated Hospital of Gannan Medical University, Ganzhou City, Jiangxi, China

**Keywords:** artificial intelligence, critical illness, bibliometric, VOSviewer, CiteSpace

## Abstract

**Background:**

Critical illness medicine faces challenges such as high data complexity, large individual differences, and rapid changes in conditions. Artificial Intelligence (AI) technology, especially machine learning and deep learning, offers new possibilities for addressing these issues. By analyzing large amounts of patient data, AI can help identify diseases earlier, predict disease progression, and support clinical decision-making.

**Methods:**

In this study, scientific literature databases such as Web of Science were searched, and bibliometric methods along with visualization tools R-bibliometrix, VOSviewer 1.6.19, and CiteSpace 6.2.R4 were used to perform a visual analysis of the retrieved data.

**Results:**

This study analyzed 900 articles from 6,653 authors in 82 countries between 2005 and 2024. The United States is a major contributor in this field, with Harvard University having the highest betweenness centrality. Noseworthy PA is a core author in this field, and *Frontiers in Cardiovascular Medicine* and *Diagnostics* lead other journals in terms of the number of publications. Artificial Intelligence has tremendous potential in the identification and management of heart failure and sepsis.

**Conclusion:**

The application of AI in critical illness holds great potential, particularly in enhancing diagnostic accuracy, personalized treatment, and clinical decision support. However, to achieve widespread application of AI technology in clinical practice, challenges such as data privacy, model interpretability, and ethical issues need to be addressed. Future research should focus on the transparency, interpretability, and clinical validation of AI models to ensure their effectiveness and safety in critical illness.

## 1 Introduction

Artificial intelligence (AI) is a broad concept that encompasses enabling computer systems to perform tasks that typically require human intelligence, such as reasoning, decision-making, or problem-solving (1). AI can be understood as an umbrella term that includes a variety of technologies such as machine learning, deep learning, natural language processing (NLP), and more (2). Over the past few years, artificial intelligence has demonstrated significant efficacy in medical diagnosis, treatment, and predictive analytics, attributed to the sophistication of its computational algorithms and machine learning capabilities (3, 4). As an innovative breakthrough in the medical field, data-driven AI technology has been widely applied in various branches of the healthcare industry, especially in the Intensive Care Unit (ICU), where it has shown great potential in handling massive and complex medical data (5, 6). For instance, in the treatment of Acute Respiratory Distress Syndrome (ARDS), by analyzing a vast amount of complex medical data, AI helps to improve the accuracy of diagnosis, predict the trend of disease development, formulate personalized treatment plans, optimize patient monitoring, guide clinical surgery and treatment decisions, and predict patients' health trajectories (7). By deeply analyzing patients' physiological data, laboratory test results, and medical history, AI can predict changes in conditions and reduce the risk of complications (8). Additionally, AI uses deep learning techniques to analyze medical imaging, assisting doctors in making more accurate diagnoses (9).

Bibliometric analysis is a method that uses mathematical and statistical techniques to qualitatively and quantitatively evaluate research in a specific field over a certain period (10). Bibliometric analysis, as a key tool, can help scholars gain insights into research trends and foresee the future direction of academic development (11). Although there is an increasing amount of research on AI models in critical illnesses, there has not yet been a systematic bibliometric review of the literature. Therefore, we conducted a bibliometric analysis to reveal the research hotspots and potential directions in the application of AI in critical illness.

## 2 Materials and methods

### 2.1 Data sources and search strategy

**Figure 1:**
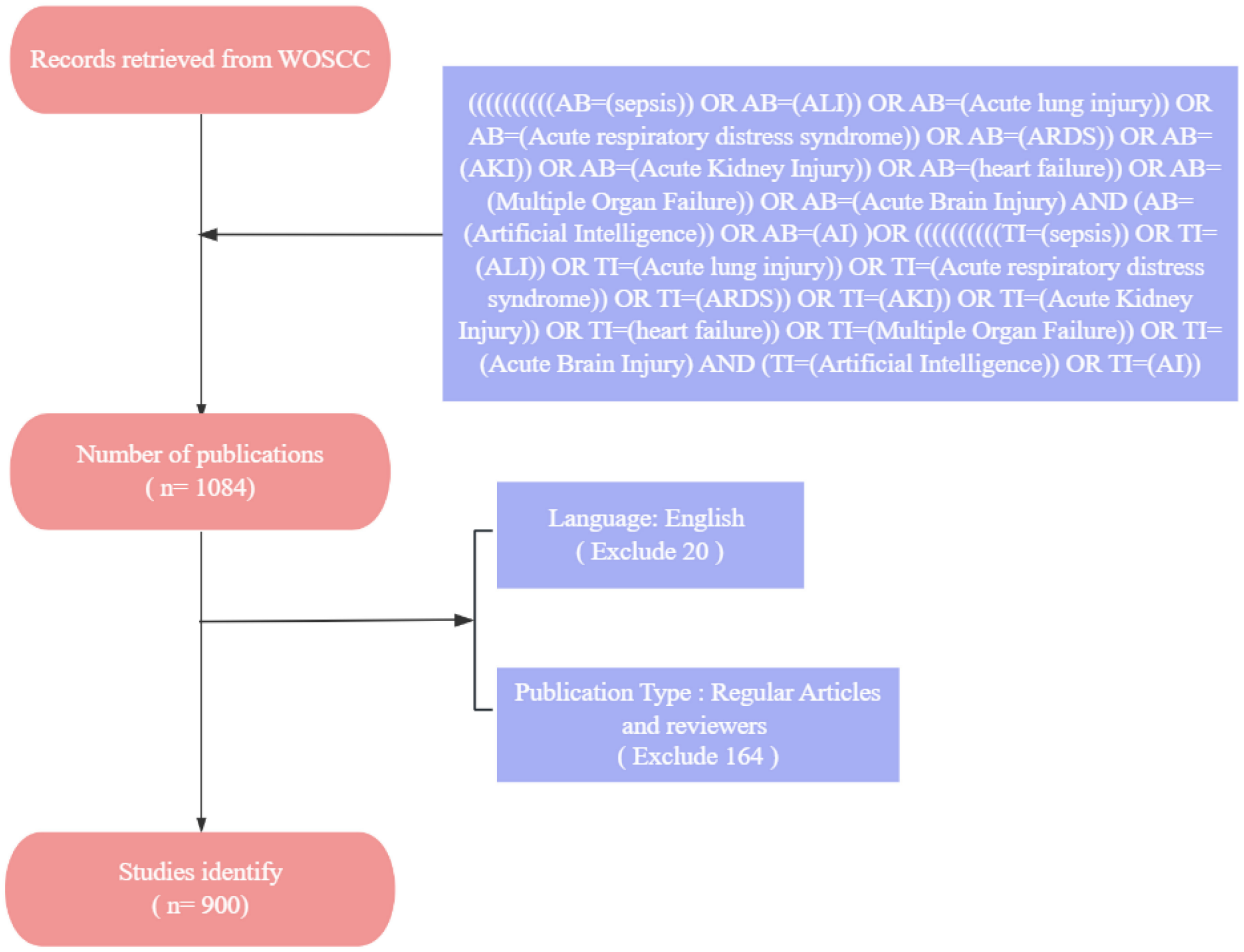
Detailed literature screening process.

For this research, we employed the Science Citation Index Expanded (SCI-E) as our main source of data. The research included in our analysis was categorized into two types: original articles and review papers, both written in English, with a time span from January 1, 2005, to September 22, 2024. To reduce the likelihood of bias from database updates, we carried out an exhaustive search and review of relevant publications, with the process being finalized on September 22, 2024. The search terms were as follows: ((((((((((AB=(sepsis)) OR AB=(ALI)) OR AB=(Acute lung injury)) OR AB=(Acute respiratory distress syndrome)) OR AB=(ARDS)) OR AB=(AKI)) OR AB=(Acute Kidney Injury)) OR AB=(heart failure)) OR AB=(Multiple Organ Failure)) OR AB=(Acute Brain Injury) AND (AB=(Artificial Intelligence)) OR AB=(AI))OR ((((((((((TI=(sepsis)) OR TI=(ALI)) OR TI=(Acute lung injury)) OR TI=(Acute respiratory distress syndrome)) OR TI=(ARDS)) OR TI=(AKI)) OR TI=(Acute Kidney Injury)) OR TI=(heart failure)) OR TI=(Multiple Organ Failure)) OR TI=(Acute Brain Injury) AND (TI=(Artificial Intelligence)) OR TI=(AI)). The specific process is shown in Figure 1.

### 2.2 Data analysis and visualization

The data extracted from the database includes the number of articles, citation counts, countries/regions, authors, journals, institutions, reference keywords, etc. (12, 13). Following that, the compiled data was then imported into Bibliometrix, CiteSpace version 6.2.R4 by Chaomei Chen of Drexel University in Dalian, China, and VOSviewer version 1.6.19 by Van Eck and Waltman from The Center for Science and Technology Studies in the Netherlands for the purpose of visual analysis. CiteSpace 6.2.R4 was utilized to analyze the citation bursts of references and keywords, and the double map overlay of journals to explore research directions and development trends (14–16). Using VOSviewer v.1.6.19, we examined the network maps showcasing the relationships and co-occurrences among authors, countries, and keywords. Bibliometrix, an open-source tool designed by Massimo Aria and Corrado Cuccurullo and based on the R language, was utilized in this study for analyzing author keyword trends and thematic topics using R version 4.3.1-win (17).

## 3 Results

### 3.1 Annual trend of publications

**Figure 2:**
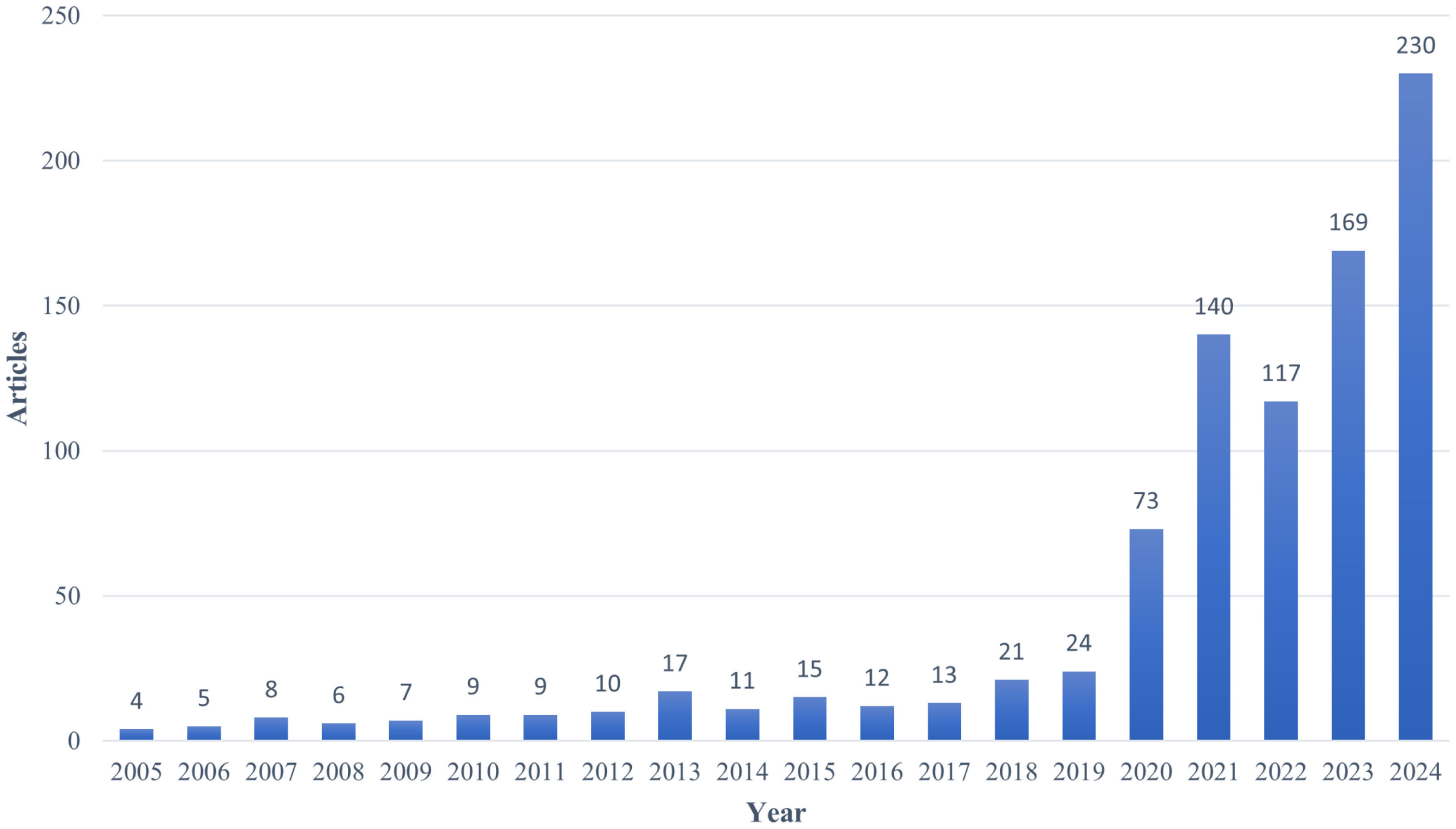
Annual output of AI in critical illness research.

A total of 900 publications were identified from 2005 to 2024. The volume of publications can to a certain degree indicate the tendencies of research within the field. As shown in Figure 2, there were few publications before 2020. With increasing attention from more researchers on the application of AI in critical illness, the number of publications in this field has increased compared to before, with a rapid growth from 73 in 2018 to 230 in 2023.

### 3.2 Analysis of countries/regions and institutions

**Figure 3:**
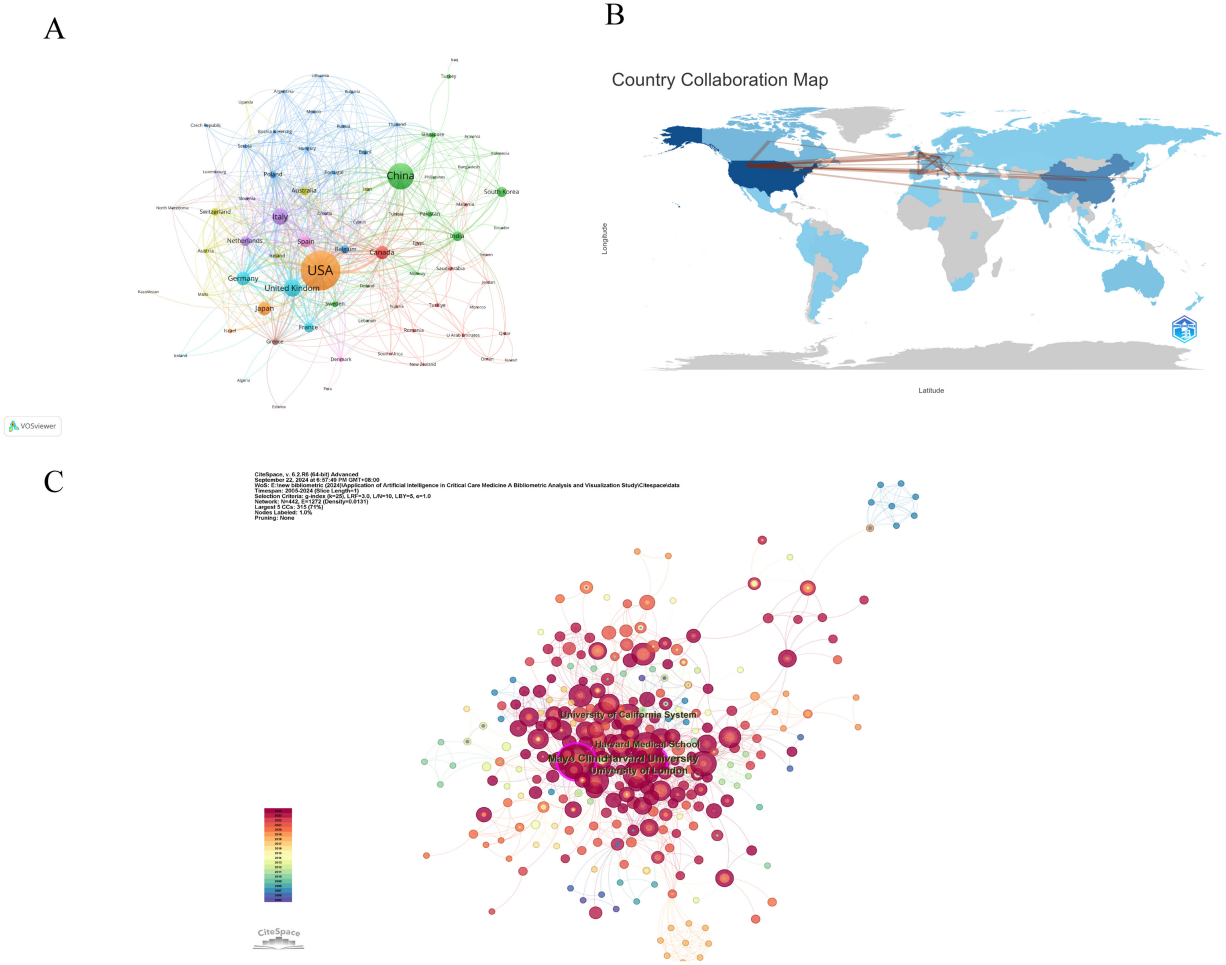
National and institutional contributions to the use of AI in critical care medicine **(A)** National co-authorship analysis map. **(B)** World map showing the total number of publications by country/region. **(C)** A co-occurrence analysis map of institutions in the field of AI in critical illness.

Table 1 ranks countries based on the publication output of authors, with the United States having the highest number of publications (*N* = 345) and citation counts (TC = 8,640). In light of collaborative relationships between countries, VOSviewer constructed a co-authorship collaboration network map among countries, with the selection criteria being countries with a publication output of at least 1 article, and 3 countries that did not have connections with other countries were removed, finally leaving 79 countries (Figure 3A). In the map, the magnitude of the nodes signifies publication counts, while the connections between nodes denote the collaborative ties between nations, with the thicker connections suggesting more robust collaborations. The United States has the most publications and the closest collaborative relationships. Figure 3B displays the global distribution of publications, with North America and Asia dominating in terms of publication output. Figure 3C is an institution co-occurrence map constructed by CiteSpace, where the size of the nodes represents the number of times an institution has been cited, and nodes with a purple halo have high betweenness centrality. The selection criteria for this map are as follows: the time span is limited to 2005–2024 with a time slice length of 1 year, and the core indicator used is the g-index (*k* = 25). In CiteSpace, a betweenness centrality exceeding 0.1 is considered a key node (18). Table 2 lists the top 10 countries ranked by their betweenness centrality. The top three institutions by betweenness centrality are Harvard University (Centrality = 0.13), Mayo Clinic (Centrality = 0.11), and University of California System (Centrality = 0.08), six of which are from the United States.

**Table 1:** Top 10 countries in terms of number of publications.

**Rank**	**Country**	**No. of documents**	**Total citations**
1	USA	345	8,656
2	China	178	2,481
3	United Kingdom	87	2,052
4	Italy	80	1,371
5	Germany	59	888
6	Japan	56	487
7	Canada	56	764
8	Spain	41	503
9	South Korea	36	548
10	Netherlands	35	784

**Table 2:** Top 10 affiliations in terms of intermediary centrality.

**Rank**	**Affiliation**	**Freq**	**Degree**	**Centrality**	**Country**
1	Harvard University	35	40	0.13	USA
2	Mayo Clinic	39	35	0.11	USA
3	University of California System	22	33	0.08	USA
4	Assistance Publique Hopitaux Paris (APHP)	17	30	0.08	France
5	University of British Columbia	13	25	0.08	Canada
6	University of London	31	31	0.07	UK
7	Icahn School of Medicine at Mount Sinai	17	28	0.07	USA
8	University System of Ohio	17	31	0.06	USA
9	Imperial College London	13	28	0.06	UK
10	Pennsylvania Commonwealth System of Higher Education (PCSHE)	17	29	0.05	USA

### 3.3 Author analysis

**Figure 4:**
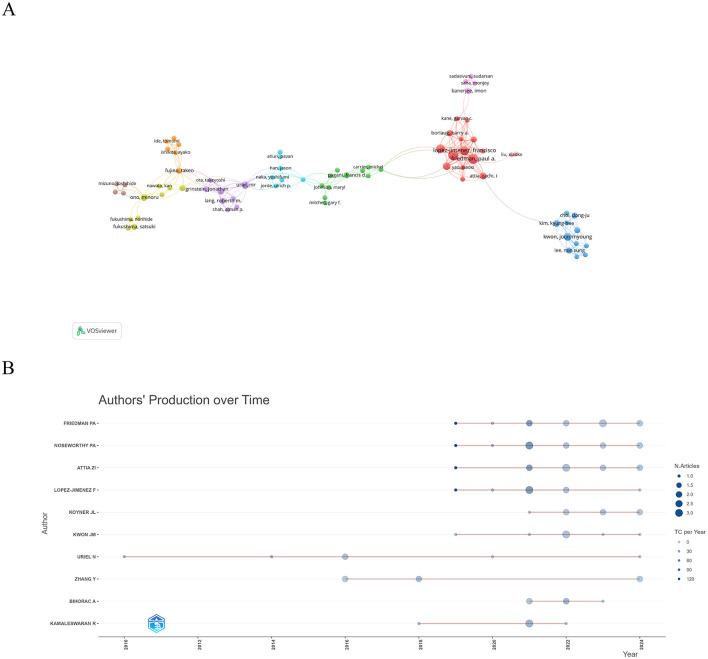
Author of a study involving the use of AI in critical illness **(A)** A co-occurrence network map of 76 authors who have co-authored two or more publications. **(B)** Productivity of the 10 most productive authors over time.

We included 76 authors who co-authored at least two publications and displayed their collaborative relationships (Figure 4A). However, it was surprising to us that there were not strong connections among these core authors, which also implies that there is a need for increased collaboration and communication among authors to promote the development of the field. Table 3 shows the top 10 authors by publication volume, with Noseworthy PA having the highest number of publications, citations, H-index, and G-index, indicating that Noseworthy PA has a significant influence in the field. Figure 4B shows the change in publications and citations over time for the top 10 authors. Notably, an article titled “An artificial intelligence-enabled ECG algorithm for the identification of patients with atrial fibrillation during sinus rhythm: a retrospective analysis of outcome prediction,” published by Noseworthy PA in 2019, has the highest number of citations (TC = 720).

**Table 3:** Top 10 authors with the highest number of publications.

**Rank**	**Author**	**Np**	**Nc**	**H-index**	**G-index**
1	Noseworthy PA	11	1,006	6	11
2	Friedman PA	11	990	5	11
3	Attia ZI	10	913	4	10
4	Lopez-Jimenez F	8	989	6	8
5	Kwon JM	7	104	4	7
6	Koyner JL	7	31	3	5
7	Uriel N	6	403	5	6
8	Zhang Y	6	82	3	6
9	Kamaleswaran R	5	152	5	5
10	Bihorac A	5	75	4	5

### 3.4 Journal analysis

**Figure 5:**
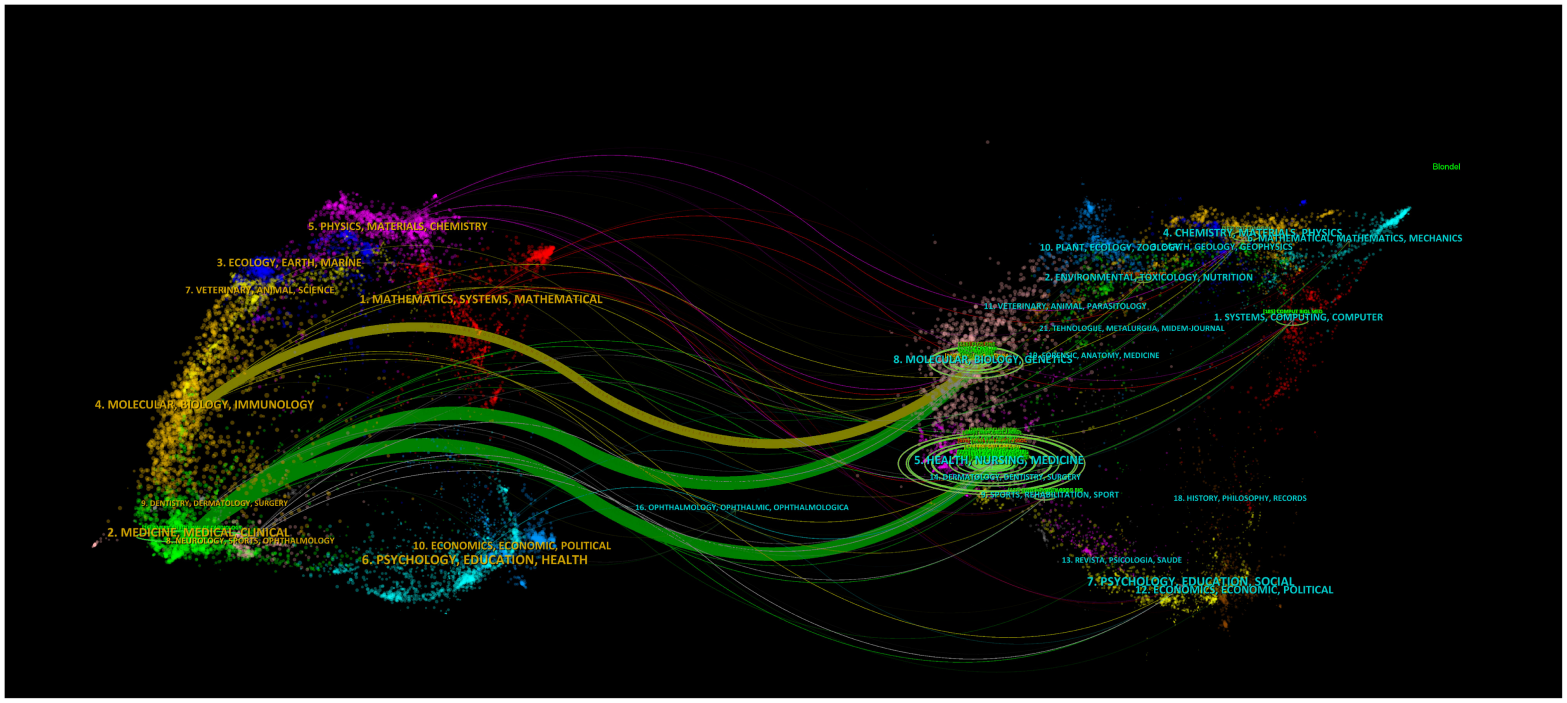
A double map overlay of AI in critical illness.

The dual-map overlay designed by Chen and Leydesdorff allows for the visualization of scientific patterns in the global scientific journal map at the disciplinary level (19, 20). CiteSpace created a dual-map overlay visualization (Figure 5) for AI applications in critical illness research spanning 2005 to 2024, with the journals that cite others on the left and those that are cited on the right. The labels indicate the academic disciplines related to the citing or cited journals, and the colored lines show the reference connections. A total of 458 journals have contributed to this field, as shown in Table 4. The journal with the highest number of publications is *Frontiers in Cardiovascular Medicine* (*N* = 23), followed by *Diagnostics* (*N* = 21). Among the top 10 journals by publication count, *the Journal of Medical Internet Research* has the highest impact factor (IF = 5.8).

**Table 4:** Top 10 journals in terms of number of articles published.

**Rank**	**Journal**	**NP**	**TC**	**H-index**	**G-index**	**IF (2024)**
1	*Frontiers in Cardiovascular Medicine*	23	126	6	10	2.8
2	*Diagnostics*	21	124	7	10	3
3	*Journal of Clinical Medicine*	17	167	6	12	3
4	*Scientific Reports*	16	222	8	14	3.8
5	*American Journal of Cardiology*	16	47	2	6	2.3
6	*Journal of Medical Internet Research*	13	401	6	13	5.8
7	*Frontiers in Immunology*	11	270	6	11	5.7
8	*Frontiers in Medicine*	10	92	5	9	3.1
9	*Journal of Cardiology*	9	5	2	2	2.7
10	*PloS ONE*	8	153	6	8	2.9

### 3.5 Co-citation bursts and citation bursts

**Figure 6:**
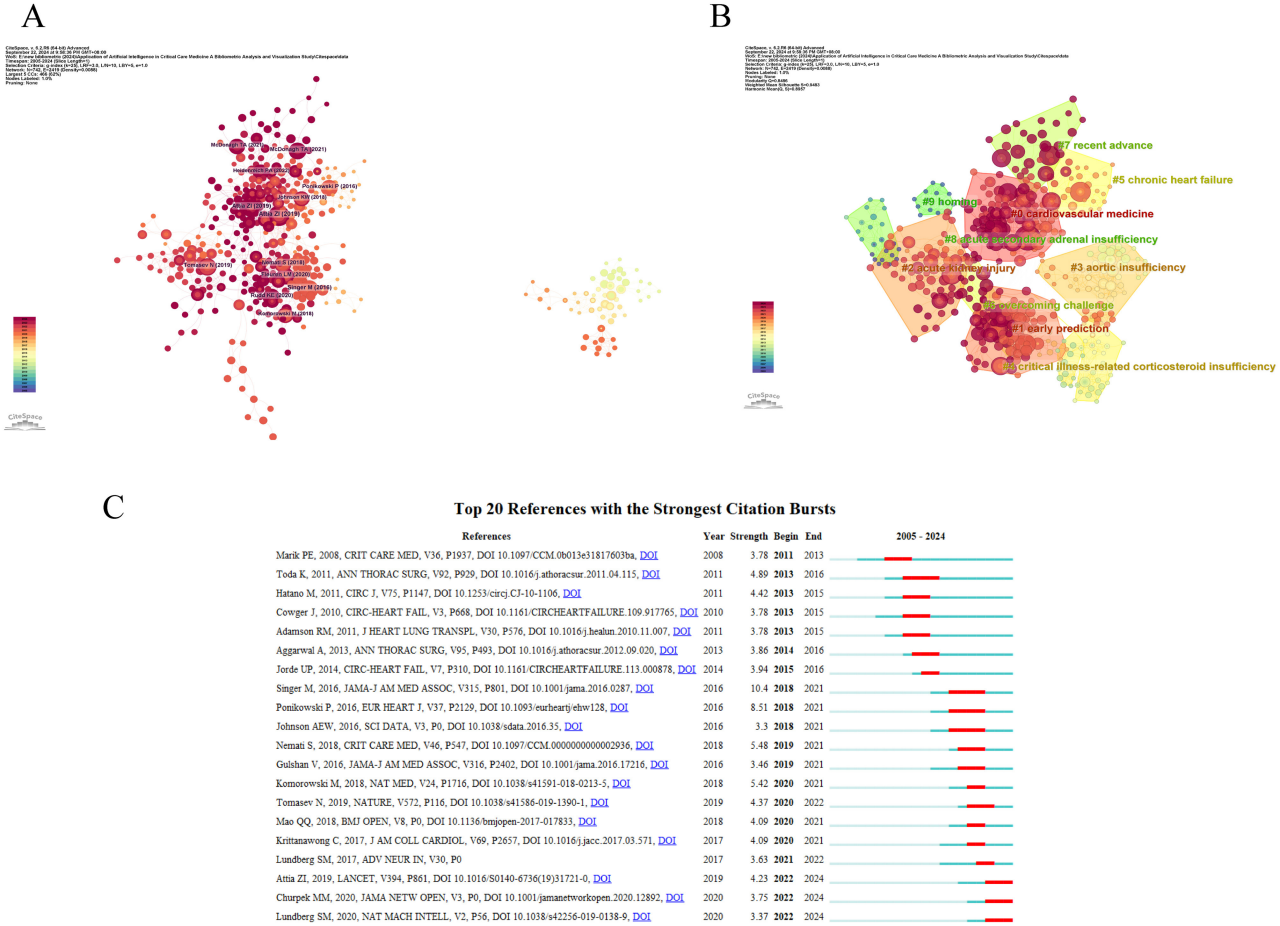
Visual map of references on the application of AI in critical illness **(A)** Schematic visualization of the reference network **(B)** Network map of reference clustering **(C)** Top 20 references with the strongest citation explosion.

In Table 5, we identified the top 10 co-cited documents by citation count. The article “Screening for cardiac contractile dysfunction using an artificial intelligence-enabled electrocardiogram,” published in Nature Medicine, has the highest number of citations. This article indicates that AI-enabled ECGs can help detect left ventricular dysfunction in asymptomatic individuals, preventing heart failure and reducing the risk of death. Figure 6A shows the visualization of citation bursts; we analyzed data from 2005 to 2024, dividing it into annual intervals to study the evolution of co-citation networks. In the CiteSpace analysis, to ensure the high credibility and academic representativeness of the clustering results, we conducted a rigorous screening of the literature, with the time span set from 2005 to 2024 and the time slice length as one year. Regarding the core indicators, we used the g-index (*k* = 25) to select high-impact literature as the basis for clustering. Based on these references, CiteSpace categorized the references into 10 clusters: #0 Cardiovascular medicine, #1 early prediction, #2 acute kidney injury, #3 aortic insufficiency, #4 critical illness-related corticosteroid insufficiency, #5 chronic failure, #6 overcoming challenge, #7 recent advance, #8 acute secondary adrenal insufficiency, #9 homing (Figure 6B). We acquired 24 reference documents with significant citation counts through CiteSpace (selection criteria: Top 20; The Number of States: 2; Minimum Duration: 2), and selected the top 20. The blue line represents the timeline, the red line represents the interval of discovery bursts, and shows the intensity of the bursts. Among them, the article titled “Internal and External Validation of a Machine Learning Risk Score for Acute Kidney Injury” has only recently begun to burst (2022–2024; Figure 6C).

**Table 5:** Top 10 most cited literature.

**Rank**	**Title**	**Corresponding author**	**Journal (IF 2024)**	**Publication year**	**Total citation (n)**
1	Screening for cardiac contractile dysfunction using an artificial intelligence-enabled electrocardiogram	Attia ZI	*Nature Medicine* (IF 58.7)	2019	40
2	An artificial intelligence-enabled ECG algorithm for the identification of patients with atrial fibrillation during sinus rhythm: a retrospective analysis of outcome prediction	Attia ZI	*Lancet* (IF 98.5)	2019	28
3	A clinically applicable approach to continuous prediction of future acute kidney injury	Tomasev N	*Nature* (IF 50.5)	2019	26
4	An interpretable machine learning model for accurate prediction of sepsis in the ICU	Nemati S	*Critical illness Medicine* (IF 7.7)	2018	25
5	Machine learning for the prediction of sepsis: a systematic review and meta-analysis of diagnostic test accuracy	Fleuren LM	*Intensive Care Medicine* (IF 27.1)	2020	24
6	Artificial intelligence in cardiology	Johnson KW	*Journal of the American College of Cardiology* (IF 21.7)	2018	21
7	The artificial intelligence clinician learns optimal treatment strategies for sepsis in intensive care	Komorowski M	*Nature Medicine* (IF 58.7)	2018	20
8	Cardiologist-level arrhythmia detection and classification in ambulatory electrocardiograms using a deep neural network	Hannun AY	*Nature Medicine* (IF 58.7)	2019	18
9	Continuous wearable monitoring analytics predict heart failure hospitalization: the LINK-HF multicenter study	Stehlik J	*Circulation-Heart Failure* (IF 7.8)	2020	14
10	Prediction of the development of acute kidney injury following cardiac surgery by machine learning	Tseng PY	*Critical care* (IF 8.8)	2020	14

### 3.6 Keyword analysis

**Figure 7:**
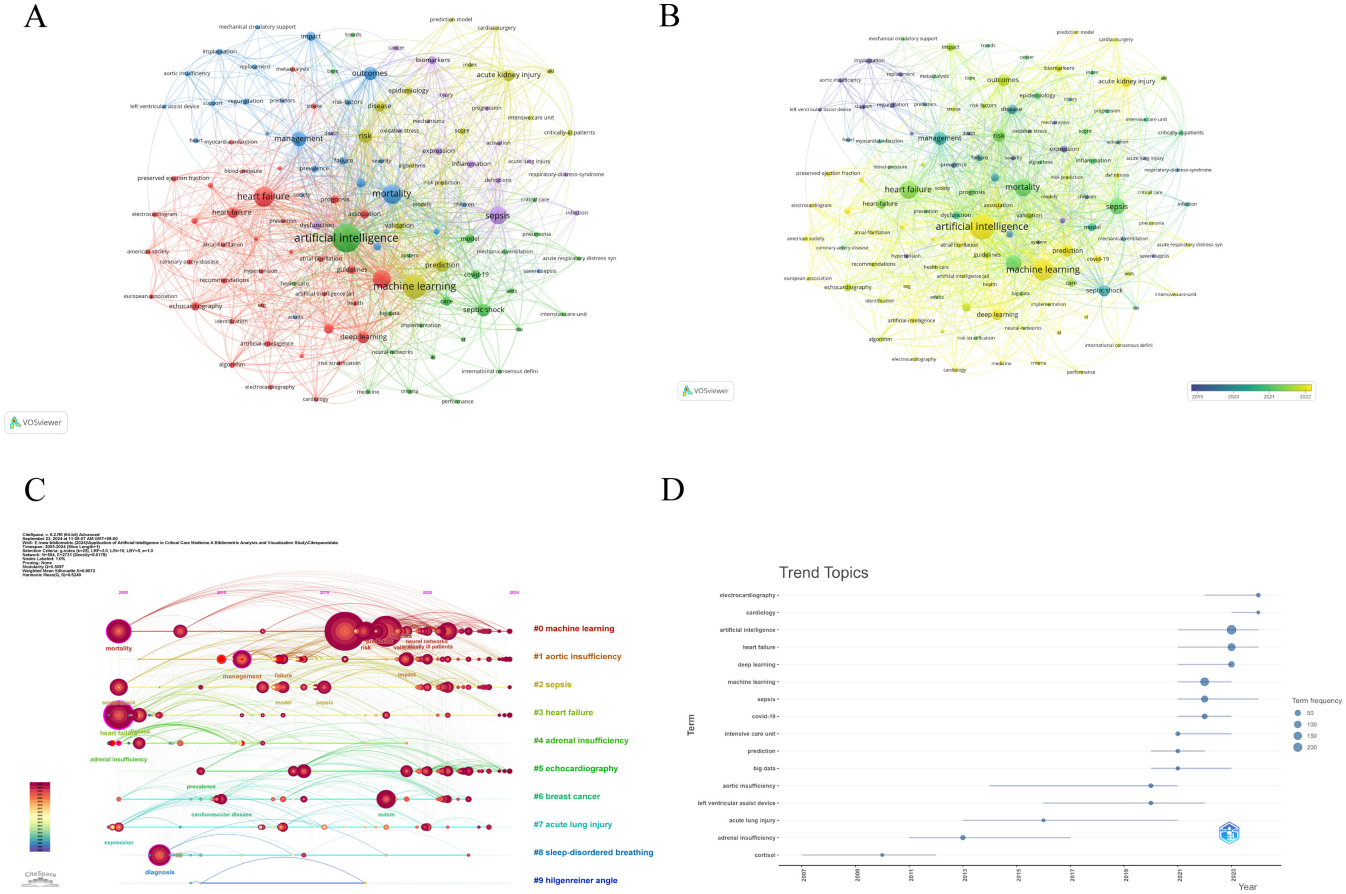
Keyword co-occurrence analysis in the application of AI in critical illness. **(A)** A visual representation in VOSviewer of 121 keywords that appeared more than 10 times. **(B)** A chronological visual map of the keywords. **(C)** A timeline graph from CiteSpace depicting the top 10 keyword clusters. **(D)** A bibliometric examination of “Trending Themes”.

Using the VOSviewer tool, we filtered out keywords that appeared more than 10 times and identified five main clusters of keywords (Figure 7A). Within this illustration, the dimensions of each node correspond to its occurrence rate, while the heaviness of the connecting lines signifies the bond's intensity; thicker lines suggest that the two keywords co-occur more often, indicating a tighter association. The five most frequently occurring keywords are: artificial intelligence (*N* = 234 times), machine learning (*N* = 173 times), heart failure (*N* = 125 times), mortality (*N* = 121 times), and sepsis (*N* = 106 times). Additionally, VOSviewer assigned colors to the keywords according to their emergence timeline, using blue for the earlier ones and yellow for those that have emerged more recently. In Figure 7B, we can see some recently emerged keywords, such as electrocardiography (2022.92), cardiology (2022.50), artificial intelligence (2022.47), and electrocardiogram (2022.41). The recent keyword trends show that the application of AI in electrocardiogram analysis, cardiology, and critical illness is becoming a hot topic of research. In particular, the application of AI technology in the automatic diagnosis of cardiovascular diseases from electrocardiograms has attracted widespread attention. This indicates that AI has tremendous potential in improving the accuracy and efficiency of diagnoses and is likely to play an increasingly important role in predicting and preventing critical illnesses. The Citespace software automatically clusters keyword and extracts the top 10 clusters. In Figure 7C, the modularity value (Q) is 0.4019, and the average silhouette value (S) of the network is 0.7283. When the Q value of the cluster exceeds 0.3, it indicates that the cluster structure is significant; an S value above 0.5 suggests that the clustering results are credible, and when the S value reaches 0.7 or higher, the clustering results are highly persuasive (3, 21). In the timeline analysis, the recently active clusters are #0 machine learning, #1 sepsis, and #2 septic shock. To better understand the trends in research topics in this field over time, bibliometric analysis was conducted for “trending topics” as shown in Figure 7D. The active keywords in recent times indicate that AI is playing an increasingly important role in the diagnosis, treatment, and prediction of cardiology, especially in the application of deep learning, showing great potential. The frequent appearance of these keywords implies the medical community's emphasis on applying AI for high-precision medical diagnosis and treatment plans, and also highlights the development trend of AI technology in improving predictive accuracy.

## 4 Discussion

### 4.1 Bibliometric information

This study used the WOSCC database to review the articles on the application of AI in critical illness over the past 20 years (2005 to 2024). The number of publications on AI in critical illness has shown an increasing trend, especially in the last 5 years, with an annual output entering a stable growth phase. The United States is in a leading position in this field, and six of the top 10 institutions by publication volume are from the United States. It is worth noting that China's publication volume in this field follows closely behind the United States, ranking second, and has a close research collaboration with the United States. This cooperative relationship not only promotes the exchange of scientific research results but also helps to advance the global application of AI in critical illness. NOSEWORTHY PA, as a researcher with significant influence in the field, has played a key role in promoting the development of the field. However, the co-authorship study reveals that the collaboration network among top AI researchers is relatively weak. To improve this situation, it is suggested to establish interdisciplinary research platforms, strengthen international cooperation projects, hold interdisciplinary seminars, share data and resources, and cultivate interdisciplinary talents. These measures will help enhance international and inter-institutional cooperation and promote further development of artificial intelligence in the field of critical illness. *Frontiers in Cardiovascular Medicine* has the highest publication volume in this field.

### 4.2 Hotspot analysis

The application of AI in the field of critical illness is developing at an astonishing rate, particularly in the areas of monitoring and diagnosis. Patients in critical condition often face multiple complications, and traditional monitoring methods are often difficult to capture subtle changes in the patient's condition in a timely manner. Studies have shown that monitoring systems using deep learning algorithms can analyze patients' physiological data in real-time, thereby providing more accurate early warnings. For example, the analysis of patient data in the ICU with the help of deep neural networks has significantly increased the early identification rate of severe infections and sepsis, which has far-reaching significance in clinical practice (22).

Bibliometric studies typically analyze the citation of references and their co-citation relationships to identify key literature, assess research progress, and predict possible future research directions. Articles with a high citation frequency are often seen as a sign of high-quality research that demonstrates significant innovation and impact within their respective fields. The analysis of these highly cited studies further emphasizes the academic impact within that particular field.

ATTIA ZI has published two highly cited articles in the field. ATTIA ZI published an article in Nature Medicine (IF = 58.7) in 2019 titled Screening for cardiac contractile dysfunction using an artificial intelligence-enabled electrocardiogram has the highest citation in the field. Among patients with asymptomatic ventricular insufficiency, those who screened positive for AI had four times the risk of future ventricular insufficiency as those who screened negative (risk ratio 4.1, 95% confidence interval 3.3 to 5.0). As a low-cost and widely available test, electrocardiography, in conjunction with AI technology, provides an effective tool for early identification of potential ventricular insufficiency in asymptomatic individuals (23). ATTIA ZI and her team published a study in 2019 in The Lancet (Impact Factor 98.5), which utilized convolutional neural networks to develop an electrocardiogram (ECG) device with integrated AI. This device is capable of detecting the electrocardiographic signature of AF during normal sinus rhythm by analyzing a standard 10-s, 12-lead electrocardiogram, thereby identifying patients with AF who are likely to develop heart failure (24). Tomasev N is also highly influential in the field, with Tomasev N and his team developing a deep learning-based model that can predict a patient's future risk of acute kidney injury (AKI) using a large electronic health record dataset in a study published in the journal Nature (25). This dataset covered patient information from 703,782 inpatients and 1,062 outpatient locations. Their model predicted episodes in 55.8% of inpatients with AKI 48 h in advance and was able to identify 90.2% of patients who required subsequent dialysis treatment. The study provided not only a confidence assessment, but also a list of clinical features that offer the potential for early intervention.

The contributions of Attia ZI and Tomasev N in the field of medical AI indicate that the field is rapidly advancing. Their research not only demonstrates the potential of AI in electrocardiogram analysis and electronic health record datasets for predicting the risk of acute kidney injury but also highlights the importance of AI technology in early diagnosis and therapeutic intervention. These research findings have increased the efficiency of disease management and may lead to new directions in future medical research, promoting the development of personalized and precision medicine.

### 4.3 Future trends

AI research is rapidly evolving in the field of critical illness, particularly in the subfields of heart failure and sepsis (Figures 7C, D). The application of AI technologies to heart failure includes early diagnosis, prognostic prediction, therapeutic decision support, and patient monitoring, whereas sepsis research focuses on early screening for diagnosis, prognostic prediction, and optimizing therapeutic management. AI technology, by analyzing a vast amount of medical data, can identify key biomarkers and patterns, thereby predicting the deterioration of conditions in advance and providing support for clinical decision-making (26).

#### 4.3.1 Application of AI in heart failure

Heart failure is a complex clinical syndrome involving various etiologies and pathophysiological mechanisms. Traditional diagnostic methods often rely on the experience and subjective judgment of doctors, while the introduction of AI technology can provide more objective and precise analysis (27). The application of AI in heart failure is prominently seen in areas such as imaging analysis, data mining, and predictive modeling, which collectively aid physicians in the early identification of diseases, the optimization of treatment plans, and the improvement of patients' quality of life. Specifically, in the realm of heart failure imaging, AI technologies are capable of processing various imaging data, including echocardiography, computed tomography (CT), and magnetic resonance imaging (MRI). These technologies can automatically detect and analyze alterations in cardiac structure and function, thereby enhancing diagnostic accuracy and facilitating timely interventions. Studies have shown that AI can play a significant role in detecting abnormalities in the left atrium and identifying potential heart issues, including atrial fibrillation. This is achieved through the analysis of ECG data, which is essential for the early diagnosis of patients at risk of heart failure (28). By leveraging AI technology, healthcare providers can enhance their ability to recognize these conditions promptly, ultimately improving patient outcomes (28). Additionally, AI can enhance the quality of cardiac imaging through image processing techniques, reduce human error, and increase the accuracy of diagnoses (29). In the diagnostic process of heart failure, the application of AI technology makes data mining and analysis more efficient. By training machine learning models, AI can extract valuable information from a vast array of clinical data, helping physicians identify high-risk patients and develop personalized treatment plans (27). For instance, research has shown that AI can predict the onset and progression of heart failure by analyzing patients' medical histories, laboratory test results, and imaging data, thereby providing support for clinical decision-making (30). AI technology has demonstrated significant potential in the management of heart failure. By continuously monitoring patients' physiological parameters in real-time, AI can aid healthcare providers in making timely adjustments to treatment plans. This proactive approach not only enhances patient care but also contributes to a reduction in both hospitalization and readmission rates. A study demonstrated that an AI-driven monitoring system is capable of accurately predicting clinical deterioration in patients suffering from heart failure, thereby alerting healthcare providers to implement timely intervention measures (31). This intelligent management model not only improves the effectiveness of treatment but also reduces the burden on the healthcare system.

#### 4.3.2 Application of AI in sepsis

Sepsis is a serious systemic infection response that often leads to multiple organ dysfunction and has a high mortality rate (32). In recent years, the swift advancement of AI technology within the medical field has introduced innovative approaches to sepsis management. By leveraging machine learning, deep learning, and other sophisticated technologies, AI is capable of analyzing extensive clinical data sets to detect early indicators of sepsis. This capability significantly enhances both the accuracy and timeliness of diagnoses, ultimately leading to better patient outcomes. The application of AI is particularly important in the early identification of sepsis. AI models can predict sepsis by analyzing patient vital signs, lab results, and other data, research shows (33, 34). This early recognition capability can significantly reduce the time to diagnosis and increase the timeliness of clinical interventions, thereby improving patient prognosis (4, 35). In addition, AI is able to help doctors develop personalized treatment plans and provide targeted advice through the analysis of historical cases to further enhance treatment outcomes (36). AI also plays a crucial role in clinical decision support for sepsis by developing intelligent systems that monitor patients' conditions in real time. These systems analyze various data points and provide treatment recommendations that align with the most current clinical guidelines and research findings, ensuring that healthcare providers have access to the best possible information for making informed decisions about patient care (37, 38). Predicting the likelihood of sepsis through AI helps focus limited resources on patients at greatest risk (39).

Machine learning, an important branch of AI, utilizes algorithms to learn from data and make predictions. Through in-depth analysis of clinical data, researchers have developed a variety of machine learning models to predict the risk of shock in patients with sepsis, and these models have shown promising results in clinical practice (40). The use of machine learning algorithms in healthcare is becoming more widespread, especially in optimizing antibiotic treatment strategies. Research has shown that these algorithms are able to analyze large amounts of data and identify key informational features in both health and disease states, thereby revealing complex interactions between treatment, patient condition, and outcomes (41). In this way, they can predict patient health trends and support clinical decision-making, particularly in the management of beta-lactam antibiotics, helping to customize more precise dosing strategies to improve treatment outcomes and reduce the risk of antibiotic misuse (41).

#### 4.3.3 Comparison with other medical AI studies

Despite the new applications of AI in critical illness revealed by keyword analysis in this study, such as heart failure, sepsis, and electrocardiogram analysis, these findings need to be compared in the broader context of medical AI development. In recent years, medical AI has made significant progress in multiple fields, including imaging diagnosis, chronic disease management, and surgical assistance (42–44). Compared with other fields, the application of AI in critical illness has unique characteristics and challenges.

The field of imaging diagnosis mainly focuses on image recognition and pattern matching, while critical illness emphasizes real-time data processing and clinical decision support. For example, deep learning is widely used in image segmentation and classification in imaging diagnosis (45), while in critical illness, it is more commonly used for the analysis and prediction of time-series data (46). This difference reflects the different needs and application scenarios of AI technology in different medical fields. Imaging diagnosis relies on high-precision image analysis to assist diagnosis, while critical illness requires rapid processing and analysis of patients' real-time physiological data to support clinical decision-making and intervention. In chronic disease management, AI technology mainly relies on data-driven intelligent algorithms to analyze and predict patients' health status and provide personalized health management plans (47). In the field of surgical operations, AI applications cover preoperative diagnosis, clinical risk prediction, intraoperative decision support, and postoperative monitoring (48).

Although the application of AI in critical illness shows great potential, its development is still in the early stage. Future research needs to further explore the commonality and specificity of AI technology in different medical fields to achieve wider application and more effective clinical practice. At the same time, strengthening interdisciplinary cooperation, promoting data sharing, and solving challenges such as data privacy and model interpretability will be the keys to promoting the widespread application of AI in critical illness.

### 4.4 Advantages and challenges of AI technology in critical illness applications

In the field of critical illness, AI technology has demonstrated significant advantages, especially in handling highly complex data, large individual differences, and rapidly changing conditions. AI technology, particularly machine learning and deep learning algorithms, can analyze vast amounts of patient data to provide more accurate diagnoses and treatment recommendations. For example, AI models can monitor patients' physiological data in real-time to predict deterioration in their condition, thereby offering timely decision support to clinicians (7, 49). Additionally, AI technology can analyze patients' medical history, laboratory test results, and imaging data to help doctors develop personalized treatment plans, thereby improving treatment outcomes.

However, the application of AI technology in critical illness also faces numerous challenges. First, data privacy is a key ethical issue. In the medical field, patients' personal information and health data are highly sensitive and must be strictly protected. The application of AI technology involves processing large amounts of patient data, which increases the risk of data breaches (50). Therefore, researchers and medical institutions must take strict measures to protect patient privacy, such as using encryption and anonymization techniques. Second, the interpretability of AI models also limits their widespread application in clinical practice. Although AI models excel at processing complex data, their “black box” nature makes it difficult for doctors and patients to understand the basis for the model's decisions (51). This not only affects doctors' trust in AI technology but also hinders its widespread application in clinical settings. Therefore, future research should focus more on the transparency and interpretability of models, using methods such as visualization techniques and feature importance analysis to reveal the decision-making process of the models (30). Finally, ethical issues are also important problems that must be faced when applying AI technology in the medical field (52). The application of AI technology must strictly comply with relevant ethical guidelines and laws to ensure the legality and morality of the research. Researchers should conduct ethical reviews before starting their research to ensure that the research design meets ethical requirements and to take measures to protect patients' personal information.

### 4.5 Limitations

Bibliometrics is a widely used tool in the academic community for assessing scientific research and academic output, but it has notable limitations. Primarily, it relies on publication counts and citation metrics to gauge research impact, which can overshadow the quality of the research itself. For instance, many high-quality studies published in lesser-known journals may not receive adequate recognition, while some studies that are frequently cited but of average quality may be perceived as more impactful than they truly are. Additionally, the fast-paced nature of research and database updates means that some new studies may be overlooked. Furthermore, in works with multiple authors, it becomes difficult to accurately evaluate each author's contribution based solely on a single, unquantified metric. Despite these drawbacks, bibliometrics can still aid scholars in quickly identifying research hotspots and development trends within their field, ultimately facilitating further exploration and inquiry.

## 5 Conclusion

In conclusion, this study highlights AI's growing role in critical illness from 2005 to 2024, with a notable increase in publications since 2020, emphasizing its importance in diagnosing and treating heart failure and sepsis. Led by the U.S., with significant contributions from Harvard University and Noseworthy PA, the field is set for expansion. Our findings underscore AI's potential to enhance diagnostics and support personalized treatments, and suggest future research should address data privacy, model interpretability, and ethics to promote AI's clinical application. We also urge policymakers to establish regulations ensuring AI's safe use. Additionally, further clinical validation of AI models is needed to improve diagnostics, personalize treatments, and strengthen patient monitoring, thus aiding AI's integration into healthcare and enhancing patient care quality.

## Data Availability

The original contributions presented in the study are included in the article/supplementary material, further inquiries can be directed to the corresponding author.

## References

[1] Collins C Dennehy D Conboy K Mikalef P. Artificial intelligence in information systems research: a systematic literature review and research agenda. Int J Inf Manag. (2021) 60:102383. 10.1016/j.ijinfomgt.2021.102383

[2] Xu Y Liu X Cao X Huang C Liu E Qian S . Artificial intelligence: a powerful paradigm for scientific research. Innovation. (2021) 2:100179. 10.1016/j.xinn.2021.100179 34877560 PMC8633405

[3] Zhao Z Hu B Xu K Jiang Y Xu X Liu Y. A quantitative analysis of artificial intelligence research in cervical cancer: a bibliometric approach utilizing CiteSpace and VOSviewer. Front Oncol. (2024) 14:1431142. 10.3389/fonc.2024.1431142 39296978 PMC11408476

[4] Yuan KC Tsai LW Lee KH Cheng YW Hsu SC Lo YS . The development an artificial intelligence algorithm for early sepsis diagnosis in the intensive care unit. Int J Med Inform. (2020) 141:104176. 10.1016/j.ijmedinf.2020.104176 32485555

[5] Chapalain X Huet O. Is artificial intelligence (AI) at the doorstep of intensive care units (ICU) and operating room (OR)? Anaesth Crit Care Pain Med. (2019) 38:337–8. 10.1016/j.accpm.2019.05.003 31102792

[6] Gutierrez G. Artificial intelligence in the intensive care unit. Crit Care. (2020) 24:101. 10.1186/s13054-020-2785-y 32204716 PMC7092485

[7] Zhang Z Navarese EP Zheng B Meng Q Liu N Ge H . Analytics with artificial intelligence to advance the treatment of acute respiratory distress syndrome. J Evid Based Med. (2020) 13:301–12. 10.1111/jebm.12418 33185950

[8] Scerri M Grech V. Artificial intelligence in medicine. Early Hum Dev. (2020) 145:105017. 10.1016/j.earlhumdev.2020.105017 32201033

[9] Rockenschaub P Hilbert A Kossen T Elbers P Von Dincklage F Madai VI . The impact of multi-institution datasets on the generalizability of machine learning prediction models in the ICU. Crit Care Med. (2024) 52:1710–21. 10.1097/CCM.0000000000006359 38958568 PMC11469625

[10] Hicks D Wouters P Waltman L De Rijcke S Rafols I. Bibliometrics: the leiden manifesto for research metrics. Nature. (2015) 520:429–31. 10.1038/520429a 25903611

[11] Wan Y Shen J Ouyang J Dong P Hong Y Liang L . Corrigendum: bibliometric and visual analysis of neutrophil extracellular traps from 2004 to 2022. Front Immunol. (2022) 13:1098082. 10.3389/fimmu.2022.1098082 36569891 PMC9774477

[12] Brandt JS Hadaya O Schuster M Rosen T Sauer MV Ananth CV. A bibliometric analysis of top-cited journal articles in obstetrics and gynecology. JAMA Netw Open. (2019) 2:e1918007. 10.1001/jamanetworkopen.2019.18007 31860106 PMC6991228

[13] Song R Guo Y Fu Y Ren H Wang H Yan H . Trends of mitochondrial changes in AD: a bibliometric study. Front Aging Neurosci. (2023) 15:1136400. 10.3389/fnagi.2023.1136400 37261264 PMC10227516

[14] Chen C. Searching for intellectual turning points: progressive knowledge domain visualization. Proc Natl Acad Sci U S A. (2004) 101:5303–10. 10.1073/pnas.0307513100 14724295 PMC387312

[15] Su Y Ruan Z Li S Li Z Chang T. Emerging trends and research foci of neuromyelitis optica spectrum disorder: a 20-year bibliometric analysis. Front Immunol. (2023) 14:1177127. 10.3389/fimmu.2023.1177127 37346048 PMC10281505

[16] Xiao Z Hu S Xu W Wang S Mo W Deng H . A bibliometric analysis of NLRP3 inflammasome in acute lung injury/acute respiratory distress syndrome from 2010 to 2021. Front Immunol. (2022) 13:1053658. 10.3389/fimmu.2022.1053658 36618363 PMC9810982

[17] Arruda H Silva ER Lessa M Proença D Jr Bartholo R. VOSviewer and bibliometrix. J Med Libr Assoc. (2022) 110:392–5. 10.5195/jmla.2022.1434 36589296 PMC9782747

[18] Chi X Fan X Fu G Liu Y Zhang Y Shen W. (2023). Research trends and hotspots of post-stroke cognitive impairment: a bibliometric analysis. Front Pharmacol. 14:1184830. 10.3389/fphar.2023.1184830 37324494 PMC10267734

[19] Zhao Y Zhu Q Bi C Yuan J Chen Y Hu X. Bibliometric analysis of tumor necrosis factor in post-stroke neuroinflammation from 2003 to 2021. Front Immunol. (2022) 13:1040686. 10.3389/fimmu.2022.1040686 36389810 PMC9661963

[20] Chen C Dubin R Kim MC. Emerging trends and new developments in regenerative medicine: a scientometric update (2000–2014). Expert Opin Biol Ther. (2014) 14:1295–317. 10.1517/14712598.2014.920813 25077605

[21] Mo W Li Q Zhou H Shi X Yang H Xiao Z . Bibliometric analysis of global research trends on pyroptosis in lung disease. Front Immunol. (2022) 13:978552. 10.3389/fimmu.2022.978552 36177039 PMC9513361

[22] Kam HJ Kim HY. Learning representations for the early detection of sepsis with deep neural networks. Comput Biol Med. (2017) 89:248–55. 10.1016/j.compbiomed.2017.08.015 28843829

[23] Attia ZI Kapa S Lopez-Jimenez F Mckie PM Ladewig DJ Satam G . Screening for cardiac contractile dysfunction using an artificial intelligence-enabled electrocardiogram. Nat Med. (2019) 25:70–4. 10.1038/s41591-018-0240-2 30617318

[24] Attia ZI Noseworthy PA Lopez-Jimenez F Asirvatham SJ Deshmukh AJ Gersh BJ . An artificial intelligence-enabled ECG algorithm for the identification of patients with atrial fibrillation during sinus rhythm: a retrospective analysis of outcome prediction. Lancet. (2019) 394:861–7. 10.1016/S0140-6736(19)31721-0 31378392

[25] Tomašev N Glorot X Rae JW Zielinski M Askham H Saraiva A . A clinically applicable approach to continuous prediction of future acute kidney injury. Nature. (2019) 572:116–9. 10.1038/s41586-019-1390-1 31367026 PMC6722431

[26] Yoon JH Pinsky MR Clermont G. Artificial Intelligence in critical care medicine. Critical Care. (2022) 26:75. 10.1186/s13054-022-03915-3 35337366 PMC8951650

[27] Yasmin F Shah SMI Naeem A Shujauddin SM Jabeen A Kazmi S . Artificial intelligence in the diagnosis and detection of heart failure: the past, present, and future. Rev Cardiovasc Med. (2021) 22:1095–113. 10.31083/j.rcm2204121 34957756

[28] Verbrugge FH Reddy YNV Attia ZI Friedman PA Noseworthy PA Lopez-Jimenez F . Detection of left atrial myopathy using artificial intelligence-enabled electrocardiography. Circ Heart Fail. (2022) 15:e008176. 10.1161/CIRCHEARTFAILURE.120.008176 34911362 PMC8766932

[29] Haq IU Chhatwal K Sanaka K Xu B. Artificial intelligence in cardiovascular medicine: current insights and future prospects. Vasc Health Risk Manag. (2022) 18:517–528. 10.2147/VHRM.S279337 35855754 PMC9288176

[30] Bourazana A Xanthopoulos A Briasoulis A Magouliotis D Spiliopoulos K Athanasiou T . Artificial intelligence in heart failure: friend or foe? Life. (2024) 14:145. 10.3390/life14010145 38276274 PMC10817517

[31] Meenakshi-Siddharthan DV Livia C Peterson TE Stalboerger P Attia ZI Clavell AL . Artificial intelligence-derived electrocardiogram assessment of cardiac age and molecular markers of senescence in heart failure. Mayo Clin Proc. (2023) 98:372–85. 10.1016/j.mayocp.2022.10.026 36868745

[32] Zeerleder S Zwart B Wuillemin WA Aarden LA Groeneveld AB Caliezi C . Elevated nucleosome levels in systemic inflammation and sepsis. Crit Care Med. (2003) 31:1947–51. 10.1097/01.CCM.0000074719.40109.95 12847387

[33] Li X Xu X Xie F Xu X Sun Y Liu X . A time-phased machine learning model for real-time prediction of sepsis in critical care. Crit Care Med. (2020) 48:e884–8. 10.1097/CCM.0000000000004494 32931194

[34] Goh KH Wang L Yeow AYK Poh H Li K Yeow JJL . Artificial intelligence in sepsis early prediction and diagnosis using unstructured data in healthcare. Nat Commun. (2021) 12:711. 10.1038/s41467-021-20910-4 33514699 PMC7846756

[35] Sullivan BA Kausch SL Fairchild KD. Artificial and human intelligence for early identification of neonatal sepsis. Pediatr Res. (2023) 93:350–6. 10.1038/s41390-022-02274-7 36127407 PMC11749885

[36] Wu M Du X Gu R Wei J. Artificial intelligence for clinical decision support in sepsis. Front Med. (2021) 8:665464. 10.3389/fmed.2021.665464 34055839 PMC8155362

[37] Schinkel M Paranjape K Nannan Panday RS Skyttberg N Nanayakkara PWB. Clinical applications of artificial intelligence in sepsis: a narrative review. Comput Biol Med. (2019) 115:103488. 10.1016/j.compbiomed.2019.103488 31634699

[38] Wang L Wu YH Ren Y Sun FF Tao SH Lin HX . Establishment and verification of an artificial intelligence prediction model for children with sepsis. Pediatr Infect Dis J. (2024) 43:736–42. 10.1097/INF.0000000000004376 38717173

[39] Hassan N Slight R Weiand D Vellinga A Morgan G Aboushareb F . Preventing sepsis; how can artificial intelligence inform the clinical decision-making process? A systematic review. Int J Med Inform. (2021) 150:104457. 10.1016/j.ijmedinf.2021.104457 33878596

[40] Wardi G Carlile M Holder A Shashikumar S Hayden SR Nemati S. Predicting progression to septic shock in the emergency department using an externally generalizable machine-learning algorithm. Ann Emerg Med. (2021) 77:395–406. 10.1016/j.annemergmed.2020.11.007 33455840 PMC8554871

[41] Ates HC Alshanawani A Hagel S Cotta MO Roberts JA Dincer C . Unraveling the impact of therapeutic drug monitoring via machine learning for patients with sepsis. Cell Rep Med. (2024) 5:101681. 10.1016/j.xcrm.2024.101681 39127039 PMC11384951

[42] Huang C Deng M Leng D Sun B Zheng P Zhang XD. MIRS: an AI scoring system for predicting the prognosis and therapy of breast cancer. iScience. (2023) 26:108322. 10.1016/j.isci.2023.108322 38026206 PMC10665820

[43] Zhou Y Chia MA Wagner SK Ayhan MS Williamson DJ Struyven RR . A foundation model for generalizable disease detection from retinal images. Nature. (2023) 622:156–63. 10.1038/s41586-023-06555-x 37704728 PMC10550819

[44] A deep learning system for predicting time to progression of diabetic retinopathy. Nat Med. (2024). 30:358–9. 10.1038/s41591-023-02742-5 38177856

[45] Tanaka R Tsuboshita Y Okodo M Settsu R Hashimoto K Tachibana K . Artificial intelligence recognition model using liquid-based cytology images to discriminate malignancy and histological types of non-small-cell lung cancer. Pathobiology. (2025) 92:52–62. 10.1159/000541148 39197433

[46] Fabregat A Magret M Ferré JA Vernet A Guasch N Rodríguez A . A machine learning decision-making tool for extubation in intensive care unit patients. Comput Methods Programs Biomed. (2021) 200:105869. 10.1016/j.cmpb.2020.105869 33250280

[47] Subramanian M Wojtusciszyn A Favre L Boughorbel S Shan J Letaief KB . Precision medicine in the era of artificial intelligence: implications in chronic disease management. J Transl Med. (2020) 18:472. 10.1186/s12967-020-02658-5 33298113 PMC7725219

[48] Zhou XY Guo Y Shen M Yang GZ. Application of artificial intelligence in surgery. Front Med. (2020) 14:417–30. 10.1007/s11684-020-0770-0 32705406

[49] Xu W Sun NN Gao HN Chen ZY Yang Y Ju B . Risk factors analysis of COVID-19 patients with ARDS and prediction based on machine learning. Sci Rep. (2021) 11:2933. 10.1038/s41598-021-82492-x 33536460 PMC7858607

[50] Martin KD Zimmermann J. Artificial intelligence and its implications for data privacy. Curr Opin Psychol. (2024) 58:101829. 10.1016/j.copsyc.2024.101829 38954851

[51] Hassan EA El-Ashry AM. Leading with AI in critical care nursing: challenges, opportunities, and the human factor. BMC Nurs. (2024) 23:752. 10.1186/s12912-024-02363-4 39402609 PMC11475860

[52] Dankwa-Mullan I. Health equity and ethical considerations in using artificial intelligence in public health and medicine. Prev Chronic Dis. (2024) 21:E64. 10.5888/pcd21.240245 39173183 PMC11364282

